# Anger Elicitation in Tonga and Germany: The Impact of Culture on Cognitive Determinants of Emotions

**DOI:** 10.3389/fpsyg.2012.00435

**Published:** 2012-10-25

**Authors:** Andrea Bender, Hans Spada, Annelie Rothe-Wulf, Simone Traber, Karsten Rauss

**Affiliations:** ^1^Department of Psychology, University of FreiburgFreiburg, Germany; ^2^Institute of Medical Psychology and Behavioral Neurobiology, University of TuebingenTuebingen, Germany

**Keywords:** culture, cognition, emotion, appraisal theory, causation and responsibility, attribution biases, universal contingency hypothesis

## Abstract

The cognitive appraisal of an event is crucial for the elicitation and differentiation of emotions, and causal attributions are an integral part of this process. In an interdisciplinary project comparing Tonga and Germany, we examined how cultural differences in attribution tendencies affect emotion assessment and elicitation. Data on appraising causality and responsibility and on emotional responses were collected through questionnaires based on experimentally designed vignettes, and were related to culture-specific values, norms, and the prevailing self-concept. The experimental data support our hypothesis that – driven by culturally defined self-concepts and corresponding attribution tendencies – members of the two cultures cognitively appraise events in diverging manners and consequently differ in their emotional responses. Ascription of responsibility to self and/or circumstances, in line with a more interdependent self-concept, co-varies with higher ratings of shame, guilt, and sadness, whereas ascription of responsibility to others, in line with a less interdependent self-concept, co-varies with higher ratings of anger. These findings support the universal contingency hypothesis and help to explain cultural differences in this domain on a fine-grained level.

## Introduction

Interactions between people of different cultural background are often strained with misunderstandings, some of which arise from diverging cognitive appraisals of the situation in which they are involved or of the event they face. Are these differences due to idiosyncratic features of the respective situation and the persons involved, or do they reflect deeper, more systematic disparities between cultures? If the latter is the case, precisely *which* cultural variables affect the way in which events are cognitively appraised and emotions elicited? And conversely, what do these differences reveal about emotions and their cognitive, social, and cultural determinants?

These questions address a core issue of cognitive science, namely how culture interacts with cognition (Bender et al., 2010; Bender and Beller, 2011b), but they also draw on appraisal theories of emotion and on attribution theory. Appraisal theories assume that emotions are elicited and differentiated by the cognitive appraisal of an event (e.g., Smith and Ellsworth, 1985; Lazarus, 1991; Frijda, 1993; Scherer et al., 2001; Fontaine et al., 2007). Appraising events in similar ways should lead to similar emotions, whereas appraising them differently should lead to different emotions. Appraising *causation* is where attribution theory enters the picture (e.g., Heider, 1958; Shaver, 1985; Hewstone, 1989; Weiner, 1995). In their attempt to find explanations for others’ (or their own) behavior, people often fall prey to attribution biases. The *self-serving bias* (Miller and Ross, 1975), for instance, leads people to take more credit for positive outcomes than for failures, thus eventually enhancing the likelihood of pride elicitation.

Appraising an event at the onset of an emotional response and attributing it to causal factors are processes which are seen *per se* as being independent of culture. Nevertheless, the ways in which these processes take place, the factors that affect them, and therefore also the results to which they lead are prone to cultural variation. The *self-serving bias*, for instance, is reinforced by the individualistic values of “Western” cultures, whereas in cultures valuing smooth interpersonal relationships, this bias is reversed (Anderson, 1999). Similarly, cultural differences in the elicitation of emotions have also been documented, both in detailed studies and large surveys (Mauro et al., 1992; Kitayama and Markus, 1994; Scherer and Wallbott, 1994; Roseman et al., 1995; Scherer, 1997a,b; Mesquita and Ellsworth, 2001). According to the *universal contingency hypothesis*, as put forward by Ellsworth (1994; and see Lazarus, 1991; Mesquita and Frijda, 1992; Scherer, 1997b), appraising events in a similar way should lead to similar emotions, irrespective of culture. That is, *if* an action is regarded a success, a likely response will be pride, but *whether* the action is regarded as a success depends on a whole range of factors, among them culture-specific concepts, values, and norms. However, there has been not much cross-cultural research on the universal contingency hypothesis. In particular, the impact exerted by culture is often not pinned down to concrete components, and systematic differences in attribution tendencies are not used to predict or explain corresponding differences in appraisal and emotions, at least not to a larger extent (cf. Oyserman et al., 2002).

Our study tries to fill this gap. It is based on the assumption that culturally defined concepts affect attribution tendencies and *thereby* alter emotional responses to given events. In order to investigate this impact empirically, we focused on the elicitation of anger and compared Germany with the Polynesian culture of Tonga, for which previous studies suggest systematic differences along crucial cultural dimensions and particularly with regard to anger elicitation (Bender et al., 2006, 2007a,b; Nerb et al., 2008; Beller et al., 2009a). After briefly depicting the theoretical and cultural background of our study, this paper presents new data that more systematically relate culture-specific concepts and attribution tendencies to emotional responses.

## Cognitive Determinants of Anger

When confronted with an event of personal relevance, people immediately, spontaneously, and for the most part unconsciously start appraising the event along several dimensions (e.g., Lazarus, 1966). While the exact nature of the appraisal dimensions and their corresponding cognitive determinants remain a subject of debate (e.g., Ellsworth and Smith, 1988; Ortony et al., 1988; Lazarus, 1991; Frijda, 1993; Scherer, 1997a, 2001; Roseman, 2001; Kuppens et al., 2003), most approaches agree that the determinants eliciting anger encompass a negative valence of the event (i.e., the degree of damage), its causation by another person, and high responsibility of this person. Results are more heterogeneous with regard to the question of whether the ability to control the event also contributes to anger and with regard to the more content-specific determinants of anger such as unfairness or immorality (cf. Ellsworth and Smith, 1988; Weiner, 1995; Roseman et al., 1996). In the context of our study, we will focus on causation (agency) and responsibility as those determinants that differentiate anger from related emotions, and we will look at damage as the determinant for the intensity of the emotional response.

### Ascribing agency

In principle, events can be caused by oneself, another person, or circumstances, and responsibility can be considered as rather high or low (e.g., Ellsworth and Smith, 1988; Roseman et al., 1996; Nerb and Spada, 2001). Although such a strict distinction is pervasive in theory, in practice the two dimensions are often conflated. If a girl drops a glass because she slips on the floor, we may still consider her as the cause, but not as responsible. In this case, low personal responsibility is not much different from circumstantial causation. Personal responsibility is discounted if the agent is not able to distinguish right from wrong or to control the respective behavior, or if he or she behaved in such a way in pursuit of a higher goal (e.g., Shaver, 1985; Weiner, 1995; Nerb and Spada, 2001).

**Figure 1:**
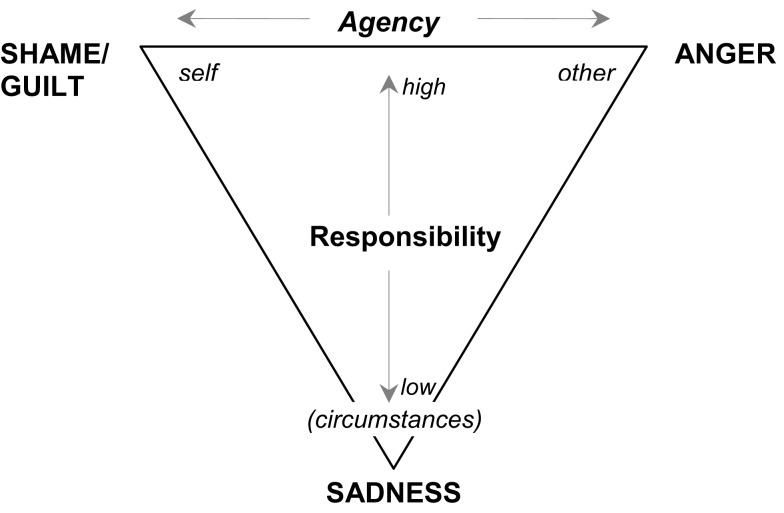
**Emotional responses to negative events varying in the source of agency and responsibility**.

Importantly, folk-psychological concepts and terminologies do not differentiate emotional responses to all combinations of causation and responsibility either. Whereas self- and other-caused events elicit clearly different emotions when high responsibility is ascribed, causation is less clearly attributed and may even be regarded as circumstantial when personal responsibility is assessed as low (see also Smith and Ellsworth, 1985). In the following, we will therefore distinguish three cases only (Figure 1): causation by other, by self (each with high responsibility), or by circumstances (low responsibility). For negative events, the emotions corresponding to the corners of this triangle are *anger* for other-caused events with high responsibility, *shame/guilt* for self-caused events with high responsibility^1^, and *sadness* for circumstances-caused events or with low responsibility.

As everyday life teaches us, people also get angry at themselves in situations in which it was them who caused the negative event. However, this *anger at oneself* was largely neglected in appraisal theories for many years. Only recently, a systematic comparison with other-anger, shame, and guilt revealed that self-anger takes a middle-position between other-anger and shame/guilt (Ellsworth and Tong, 2006). In particular, self-anger differs from other-anger in appraisals of causation, fairness, and control, and it differs from shame/guilt in appraisals related to the obstacle and its moral value, and in the actual feeling of boiling inwardly. While these findings provide a detailed characterization of self-anger in the US, the question of whether self-anger occurs cross-culturally remains open so far.

Cross-cultural studies (e.g., Ben-Zur and Breznitz, 1991; Mauro et al., 1992; Roseman et al., 1995; Scherer, 1997a,b; Gidron et al., 1998; and see Mesquita and Ellsworth, 2001, for an overview) provide evidence for similar links between such sets of determinants and specific emotions across cultures, particularly for basic emotions like anger, sadness, and shame/guilt. But they also suggest cultural differences in appraisal tendencies that hinge, to a considerable extent, on the way in which causation is attributed and responsibility is ascribed.

### Attribution tendencies and anger

Although not all appraisal processes require the identification of a specific cause, some do – particularly those involved in eliciting the “attribution emotions” like pride, shame, or anger (Ortony et al., 1988). However, appraisal theories and attribution theory differ with regard to the number of causes to be distinguished. Most appraisal theories typically differentiate three sources of causation, whereas attribution theory focuses on two types of attributions only: one primarily referring to the person and his or her dispositions (internal attribution) and one primarily referring to the situation (external attribution). In order to integrate these diverging causal conceptions of attribution theory and appraisal theories, one can take advantage of the differences in perspective between actor and observer. Following Watson (1982), who rephrased this distinction in terms of self and other, we can relate the terminology of attribution theory more closely to that used in appraisal theories, with self vs. other as endpoints of the *agency* dimension, and internal vs. external locus (i.e., self/other vs. circumstances) as endpoints of the *responsibility* dimension (see Figure 1).

As accurately assessing causation requires cognitive effort and attention, people may fall prey to a range of biases when making attributions. Among the most important biases are the *actor observer difference* (Jones and Nisbett, 1972; Watson, 1982), the *correspondence bias* (formerly called fundamental attribution error; Ross, 1977; Jones, 1990; Ross and Nisbett, 1991; Gilbert and Malone, 1995), and the *self-serving bias* (Miller and Ross, 1975). These attribution tendencies have consistent effects, not only with regard to the ascription of responsibility, but also – as part of the appraisal process – with regard to the emotional responses: for negative events, they should generally *increase* both the ascription of responsibility to others and, consequently, the likelihood of an angry response, and they should *decrease* both the ascription of responsibility to self and the likelihood of shame/guilt. However, these attribution tendencies do not occur universally, but appear to be systematically affected by culture.

### Cultural differences and consequences

The majority of cross-cultural studies concerned with attribution and appraisal processes identify cultural differences along one dimension each: individualistic vs. collectivistic values (Hofstede, 1980; Triandis, 1995), an independent vs. interdependent self-concept (Markus and Kitayama, 1991), implicit theories of individual vs. group agency (Menon et al., 1999; Morris et al., 2001), or analytic vs. holistic systems of thought (Norenzayan and Nisbett, 2000; Nisbett et al., 2001). Despite differences within and across each of these traditions, all of them share an emphasis on the relation between individual and group (for a synthesis, see Peng et al., 2001; for an alternative approach see Fiske, 1992). In recent years, parts of this cross-cultural approach have been criticized from various perspectives for theoretical, conceptual, and methodological reasons (e.g., Takano and Osaka, 1999; Fiske, 2002; Oyserman et al., 2002). We took these critiques seriously and therefore developed our research from sound anthropological fieldwork.

#### Culture-specific self-concepts and attribution tendencies

Self-concepts are among the most extensively investigated and documented modulators of responsibility ascription. Their two dimensions *independence* and *interdependence*, although not entirely mutually exclusive, focus on diverging aspects. A more independent self-concept is typically emphasized in “individualistic” cultures in which self-esteem is supported, personal accomplishments are important for one’s identity, and rights are valued over duties. In more “collectivistic” cultures, on the other hand, the interdependent aspects of the self are emphasized. People are seen as parts of larger social groups that bind and mutually obligate them, social harmony is of prime concern, and duties are valued over rights (Hsu, 1981, 1983; Markus and Kitayama, 1991; Triandis, 1995).

These cultural differences should have implications for attributions. And indeed, various comparisons between US Americans, prototypical of individualistic Western cultures (Hsu, 1983), and members of the more collectivistic East Asian cultures like China reveal important differences. Americans tend to overestimate the role of dispositional factors in defining behavior and therefore ascribe higher personal responsibility to the actor (i.e., the other) than Asians, who are apparently more willing to take information about situational influences into account (e.g., Miller, 1984; Morris and Peng, 1994; Morris et al., 1995; Choi and Nisbett, 1998). The latter effect may result from attributing to a different entity, namely to groups instead of individuals (Menon et al., 1999), but the relevant point remains: with regard to individuals, attribution styles diverge significantly – the correspondence bias is less pronounced in collectivistic countries like China than in individualistic Western countries.

In a similar manner, maintaining and enhancing one’s self-esteem is an appropriate matter of concern in individualistic cultures, thus paving the way for the self-serving bias. The reversed pattern is found in collectivistic cultures, where smooth interpersonal relationships are more important than self-esteem (e.g., Anderson, 1999).

#### Implications for emotions

If we assume that attributing causation and ascribing responsibility are crucial for the elicitation and differentiation of specific emotions, then cultural differences in attribution styles should lead to different emotional responses, at least in terms of intensity (e.g., Ellsworth, 1994; Scherer, 1997b; Mesquita and Ellsworth, 2001; Mesquita and Markus, 2004). Accordingly, a divergence in these biases should entail a divergence in emotional responses. More precisely, the correspondence bias, being more pronounced in individualistic cultures, should result in ascribing higher responsibility to others, thereby also enhancing anger in negative events. In collectivistic cultures, on the other hand, personal responsibility and the subsequent emotional responses should be reduced (for effects of the self-serving bias see Anderson, 1999; Bender et al., 2006).

Besides affecting the elicitation of emotions, attribution tendencies could also affect the way in which emotions themselves are attributed to other people. Given that many emotions are linked to cultural values and some are even socially sanctioned, self- and other-serving biases may affect the readiness with which people perceive these emotions in themselves or others. However, to the best of our knowledge, this type of impact is yet to be investigated.

And finally, the degree of perceived negative valence (or “damage”), which accounts for the intensity of the experienced emotion, partly depends on cultural definitions of what counts as negative in the first place. Norm violations, for instance, will only be regarded as negative if the violated norm and the underlying values (e.g., social harmony or self-realization) are shared in the respective culture.

## Values, Self-Concept, and Emotions in Tonga and Germany

The purpose of our paper is twofold: we are trying to find an explanation for apparent differences in emotional – and particularly angry – responses to similar events across cultures (i.e., between Germany and Tonga), and we are trying to empirically corroborate our conjecture that these differences are brought about by differences in attribution styles, triggered by culture-specific concepts and values.

The choice of Tonga for the comparison is justified by several reasons: the small Polynesian kingdom with its venerable history (Campbell, 2001) is renowned for its cultural resilience and the strong cohesion of its core social units. Its cultural context is clearly distinct from both the holistic tradition of East Asian philosophy and the analytic tradition of Western philosophy (Nisbett et al., 2001), and therefore provides an interesting extension to the more common American-Asian comparisons^2^. Extensive anthropological fieldwork in Tonga, much of it conducted on indigenous folk psychology, values, and emotions (e.g., Marcus, 1978; Bernstein, 1983; Martin, 1991; Morton, 1996; Bender, 2001, 2002; Evans, 2001; Bender and Beller, 2003; Bender et al., 2007a,b) allows us to integrate our experimental data into the broader context of Tongan culture, with its emphasis on social relationships, appropriate behavior in interactions, and the control of specific emotions.

Tongan society is hierarchically structured with asymmetrical relationships according to rank and status originating from within the nuclear family. Linked with these differences in rank are social rules of respect and obedience. Consequently, the father’s sister or *mehekitanga* – as the highest-ranking person in one’s family – is entitled to request all kinds of support, and obeying these requests is regarded not only obligatory, but also an expression of respect. Disrespectful behavior (particularly vis-à-vis a person of higher rank) is thus among the most frequent causes for anger, followed by violations of justice and fairness, but also events that, in one way or another, curb participants’ personal freedom (Bender et al., 2007a).

In contrast, the traditional German values discipline, obedience, or tidiness are increasingly displaced by values that focus on self-realization such as independence, self-confidence, the abilities to judge and to assert oneself (Pross, 1982), supplemented by the general wish of having a good life (Noelle-Neumann and Petersen, 2001; Bender et al., 2007a). Potential causes for anger elicitation therefore include those factors experienced as obstructing own goals, such as incompetence, unpunctuality or unreliability, but also the violation of norms of justice and fairness.

In Tonga, people are to a large degree determined in their options and activities by other members of their social network. Whereas such an experience may be regarded as negative by Western (i.e., individualistic) standards, the willingness to help and share and the strong social support that come with it are highly valued by most Tongans. Such attitudes are regarded as proper expressions of *‘ofa*, which is the core value in Tongan culture. *‘Ofa* – roughly to be summarized as “love, fondness, kindness,” but also referring to concern, care, help, and generosity – characterizes the ideal emotional relationship between people and is “the philosophy behind their way of life” (Kavaliku, 1977, p. 67). As social harmony is particularly emphasized, socially disruptive emotions like anger and open conflicts are disapproved of.

If we relate these observations to the core theoretical constructs of cross-cultural research, we may infer that people in Tonga value interdependence more than independence and particularly more than Germans do. Previous studies support this assumption, revealing a significantly stronger interdependent self-concept for Tongans than Germans (and even Chinese), whereas the independent aspects of the self are rated rather similarly^3^ (Beller and Bender, 2004; Bender et al., 2006; Beller et al., 2009a). However, previous studies also showed that differences in self-concept must not be regarded as the *only* factor relevant for differences in causal attribution and responsibility ascription. For instance, a study on how causal roles are assigned in the physical domain demonstrated the relevance of linguistic factors and culture- (and domain-) specific concepts (Beller et al., 2009b; Bender and Beller, 2011a), and a systematic variation of situations involving social inducements points toward situational aspects as at least as, if not more, influential than differences in self-concept (Beller et al., 2009a).

Empirical studies based on vignettes also support our assumption that the two cultures differ with regard to attribution tendencies. In response to a questionnaire that contrasted positive and negative events, German and Tongan participants alike are more ready to take responsibility for good deeds than for bad. However, their ascriptions of responsibility to *others* diverge in interesting ways: Germans do so to a greater degree for bad deeds, while Tongans do so for good deeds. This corresponds to a strong self-serving bias in Germany, eliciting relatively little shame for bad deeds, but a great deal of pride for good ones. The reversed attribution tendency in Tonga eclipses the initial self-serving bias and produces a high degree of shame for bad deeds and a lower degree of pride for good ones (Bender et al., 2006).

Encouraged by these results from previous studies, we set out to extend our analysis of how culture and cognitive processes interact in shaping emotions, focusing now on the correspondence bias and the conditions under which anger is elicited – and elicited differently in the two cultures. In this paper we present data from a new experiment that was conducted with a larger number of participants and that more systematically varied causes and contents. As we are particularly interested in principles that affect the elicitation of anger, the experiment was restricted to negative events and to anger and some of its conceptual “neighbors” along the attributional dimensions.

In order to test our hypotheses, two sets of vignettes were used that systematically vary content and causation, respectively. For the first set, we chose instances of norm violations and varied the degree to which the respective norm is relevant in the two cultures. We expected to find differences in the appraisal of damage, corresponding to the salience of the violated norm, and respective differences in the intensity of specific emotions. The second set depicted negative events in which a personal (though socially oriented) goal is obstructed and the source of causation is explicitly varied: by oneself, another person, or circumstances. Here, we expected Tongans to ascribe responsibility for these events more evenly to the three causes than their German counterparts. Again, emotional responses should reflect these patterns of attributions.

## Materials and Methods

In order to ensure a valid choice of terminology and scenarios, the construction of the experimental material was based on extensive anthropological fieldwork in Tonga by the first author, which consisted of participant observation, informal talks, interviews, pile sorting tasks, and linguistic analyses (for more details see Bender et al., 2007a). This paper focuses on a study based on questionnaires with experimentally varied vignettes and ratings for the frequency of emotions. The respective material was the first part of a larger survey; only those parts of the material and results relevant to our current questions are reported here.

### Participants

For theoretical and methodological reasons, we recruited students from higher years of secondary schools^4^. The German sample consisted of 254 students from a high school in Siegen (121 male, 131 female, 2 did not indicate their gender), with a mean age of 14.8 years (*range*: 13–19 years). The Tongan sample consisted of 65 students from a high school in Pangai (35 male, 29 female, 1 did not indicate his or her gender), with a mean age of 15.7 years (*range*: 13–18 years). Participants were recruited in the classroom and rewarded by contributions to their class funds. Due to missing values, some participants had to be excluded, but in order to retain as many participants as possible the exclusions were conducted separately for each calculation.

### Material

Data was collected with different tools: one scale for assessing the self-concept, one emotion frequency questionnaire, and one questionnaire based on experimentally varied vignettes.

#### Self-concept scale

To assess self-concept, we used the *Self-Construal Scale* of Singelis (1994) in a slightly shortened version (two inconsistent items in each subscale were eliminated in order to enhance reliability^5^). Participants were instructed to rate the degree to which each statement applied to them on a five-point scale ranging from 0 (“not at all”) to 4 (“completely”).

#### Emotion frequency questionnaire

Participants also had to assess the frequency of a range of emotions. For exploratory purposes, the list contained 19 different emotions, including both positive and negative ones. Among the emotions were anger, anger at oneself, shame, guilt, and sadness. The questionnaire was constructed in two versions and administered in a between-subjects design. The self-referenced version asked participants to state how often they themselves had experienced the respective emotions within 1 week, while the other-referenced version asked them to state how often their classmates had experienced these emotions. Again, frequencies were rated on a five-point scale ranging from “very rarely” to “very often.”

Please note that we did *not* intend to rely on the actual values of reported frequencies, as these may be severely affected by memory biases (Levine et al., 2006), but were primarily interested in differences between the self- and other-referenced versions.

#### Vignettes

The vignettes consisted of two sets of scenarios, each followed by a range of questions. The scenarios were constructed as follows: first, we collected events both in Germany and Tonga that elicited negative emotions by way of emotion diaries in which participants were also requested to specify details of the emotion-eliciting events (Bender et al., 2007a). Based on these events, we derived scenarios and systematically varied them for each triplet.

In the first triplet, content was varied. Each story portrayed an interaction between two people, one of whom is violating a specific norm. The norms are: respecting one’s father’s sister (TN = *Tongan norm*), being punctual (GN = *German norm*), and judging fairly (SN = *shared norm*). Two of the scenarios were constructed so as to elicit anger asymmetrically: scenario TN deals with a norm specific to the Tongan culture (where the father’s sister is the highest-ranking person in a family), GN with a norm specific to Germany. Finally, the SN scenario deals with a norm shared by members of both cultures and is supposed to elicit anger symmetrically in both cultures. As we are particularly interested in the role of responsibility, the scenarios make clear the negative outcome and depict the other person as *causing* the damage, but personal *responsibility* can be attributed differently. The phrasing of the scenarios runs as follows:

TN: Mary is David’s aunt, and she lives in the same road as David’s family. Mary instructs David to go shopping for the party she is planning. David tells his aunt that he can’t do it.
GN: Peter and James want to meet up. However, James arrives almost one hour later than they have arranged.
SN: During a ball game, John is very committed. When attempting to score a decisive point, John is fouled by a player of the opposing team. The referee lets them continue their game.

The second triplet varied the sources of causation (OC = *other-caused*, SC = *self-caused*, CC = *circumstances-caused*). Two sources of causation (i.e., other and self) correspond to high personal responsibility, while the third (i.e., circumstances) corresponds to low personal responsibility^6^. While the negative outcome is stated and causation is varied, personal responsibility can be attributed differently. The phrasing of the scenarios runs as follows:

OC: Tom has just bought a gift and is walking down the street with it. Suddenly he is pushed by a boy. The gift falls to the ground and breaks.
SC: Tom has just bought a gift and is walking down the street with it. Suddenly he stumbles. The gift falls to the ground and breaks.
CC: Jane has organized a big party outside to which she has invited many guests. Everything is prepared and festively decorated and all of the guests have arrived, when suddenly a storm breaks. The party falls through.

Each story in each triplet was followed by several questions. The first question asked participants to take the perspective of the affected persons and to rate their emotional responses in the event (e.g., “Which feelings will Peter have to what extent because James arrives almost one hour later than they have arranged?”). A multiple-choice format was used, with 11 emotions covering an array of negative states, among them anger (German: *Ärger*, Tongan: ‘*ita*), sadness (*Traurigkeit*, *loto-mamahi*), shame (*Scham*, $m\bar{a}$), and guilt (*Schuld*, *loto-tautea*)^7^. For the second triplet with causation varied, anger at oneself (*Ärger über sich, ‘ita ‘ia kita pē*) was added. For each emotion, participants had to indicate its intensity on a five-point scale ranging from “not at all” to “very.”

Subsequent questions asked for an assessment of how severe (*schlimm, kovi*) the specific incident is (i.e., negative valence or damage) and of how responsible other, self, and circumstances are for the specific incident. Again, participants had to indicate their ratings on a five-point scale ranging from “not at all” to “very.”

All materials were presented in the participants’ native language and used customary names for the persons involved. Translation took place through English and was conducted by one German/English bilingual person, two English/Tongan bilingual persons, and one person with fluency in German, English, and Tongan. The translations were double-checked by native speakers and pre-tested to ensure comprehensibility. A larger range of Tongan emotion terms was elicited with free-listing and collected from interviews and daily conversations in Tonga. Those used in our study were selected after semantic tests and consultation with native speakers of Tongan^8^. Particular care was taken concerning anger, the emotion of prime interest in our study. In order to combat reservations by a line of research that emphasizes the incommensurability of emotion terms across languages (e.g., Lutz, 1982, 1988; Wierzbicka, 1999; Durst, 2001; Harkins and Wierzbicka, 2001), we included in our preparatory work a systematic comparison of the different components of the anger sequence that established sufficient similarity between the emotional cores of German *Ärger* and Tongan *‘it*a (Bender et al., 2007a).

Typicality of all scenarios was checked by asking participants (*n* = 21 high school students in Tonga and 39 in Germany) in an otherwise similarly designed pilot study how typical they considered the depicted scenarios. No significant differences were found between cultures. Usability of the rating scales was also pre-tested in Germany and Tonga.

### Procedure

Data collection took place in the classrooms. Each participant received a booklet with general instructions, the vignette questionnaire, the scale, and the frequency questionnaire (in this order). The scenarios in each triplet were presented within subjects, with the first triplet first, followed by the second. Within triplets, the order of the vignettes was randomized to control for order effects. Each story began on a new page; the questions and answering options were presented in the same order as described above. Participants were instructed to answer all questions in the given order, and were granted as much time as they needed.

### Hypotheses

The general idea is based on the observation that, on certain occasions, Tongan and German emotional responses differ markedly. The main purpose of our study is therefore to identify content-specific causes for these differences. Specifically, we expected that cultural differences in emotional responses (1) depend on the content of events, but (2) may also arise from differences in attribution processes, in line with the prevailing self-concept. Hypothesis (1) thus states that the more salient a violated norm in a given culture, the greater its assessment as damage and the more intense the emotional response. Hypothesis (2) states that the less interdependent the self-concept, the more prone to the correspondence bias (which reduces likelihood to take circumstances into account), and the more likely to respond with anger.

In addition, the status of anger at oneself as a potential response to negative events will be addressed. Here, we assume patterns largely similar to those for shame and guilt, namely high ratings for events caused by self and low ratings for other-caused events.

### Data analysis

Data were analyzed using SPSS versions 15 and 17. For repeated-measures Analysis of variances (ANOVAs), multivariate results will be reported in detail where appropriate, and the results of follow-up univariate analyses will be described briefly in the text, with details provided in tables. These results are reported to provide for an overview of the cross-cultural differences and similarities in appraisal dimensions and emotional responses. However, given our hypothesis on the culture-specific, indirect effects of the different scenarios on participants’ emotional responses via appraisal dimensions, we additionally conducted analyses of moderation and mediation effects, as originally described by Baron and Kenny (1986) and later adapted to within-subjects designs (Judd et al., 2001) and multiple mediator models (Preacher and Hayes, 2008; more details on this are provided in Appendix).

## Results

### Self-concept in Tonga and Germany

In line with our previous studies (Beller and Bender, 2004; Bender et al., 2006; Beller et al., 2009a), we expected the Tongan students to be more interdependent than the German students (whereas independence was never found to produce significant differences). This pattern was replicated in the present study. ANOVA of the two subscales of the Self-Construal Scale (Singelis, 1994) showed a significant intercultural effect for interdependence [*F*(1, 266) = 132.69, *p* < 0.001, partial η^2^ = 0.33], with higher values observed in Tonga (*M* = 3.11, *SD* = 0.57) than in Germany (*M* = 2.18, *SD* = 0.55). With regard to independence, the two cultures did not differ (Germany: *M* = 2.50, *SD* = 0.56; Tonga: *M* = 2.48, *SD* = 0.60; *F* < 1). The results thus confirmed that our sample of Tongan participants is more interdependent than the German sample, which is a necessary precondition for testing our main hypotheses.

### Emotion frequencies for self and other

In order to obtain some evidence on how much our experimental data may be affected by social desirability, our participants were requested to assess the frequency of a range of emotions in either a self-referenced or other-referenced version. In view of the specific evaluation of emotions in the two cultures, we assumed that socially disruptive emotions such as anger should be reported more frequently in Germany than in Tonga, whereas the reverse should hold for conciliatory emotions (and particularly shame and guilt). In particular, the underlying cultural evaluations of emotions should contribute to an impact of culture-specific attribution tendencies on the attribution of emotions. If this assumption holds, then our German participants should – due to the self-serving bias – attribute negative emotions more readily to others than to themselves, whereas the reverse should take place in Tonga ensuing from the reversed self-serving (i.e., other-serving) bias.

The frequency data for the relevant emotions anger, anger at oneself, shame, sadness, and guilt were analyzed by multivariate analysis of variance (MANOVA) with culture and reference (self vs. other) as between-subjects factors. Results showed a significant overall effect of culture [*F*(5, 273) = 16.86, *p* < 0.001, partial η^2^ = 0.24] as well as a significant culture × reference interaction [*F*(5, 273) = 4.55, *p* = 0.001, partial η^2^ = 0.08]. By contrast, there was no significant overall effect of reference (*F* < 1).

**Figure 2:**
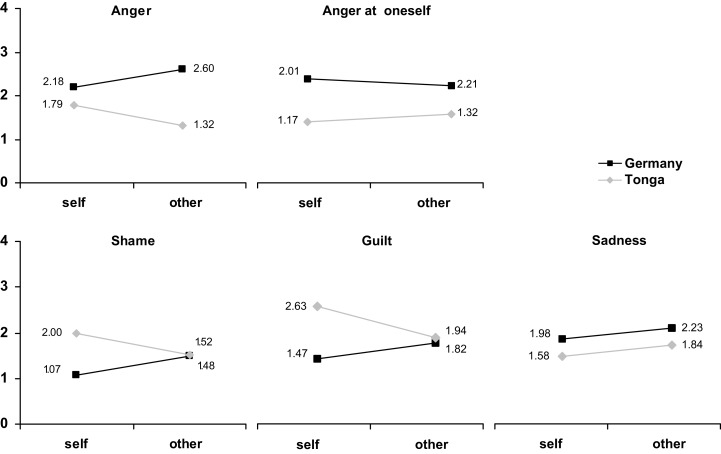
**Reported frequency ratings for anger, anger at oneself, shame, guilt, and sadness as a function of culture and reference (self vs. other)**.

Subsequent univariate analyses (Table 1) demonstrated culture effects for all five emotions: values for anger, anger at oneself, and sadness were higher in Germany, whereas ratings of both shame and guilt were higher in Tonga. Interaction effects were limited to anger, shame, and guilt (Figure 2).

**Table 1:** **Univariate effects of reported emotion frequencies**.

	*M* *(SD)_G_*	*M* *(SD) _T_*	*F*	*df*	*p*	*part*. η^2^
**CULTURE EFFECTS**						
Anger	2.37 (1.00)	1.53 (1.26)	28.00	1, 277	<0.001	0.09
Anger at oneself	2.10 (1.20)	1.25 (1.22)	22.52	1, 277	<0.001	0.08
Shame	1.25 (1.01)	1.73 (1.28)	9.42	1, 277	0.002	0.03
Guilt	1.63 (1.19)	2.24 (1.31)	12.31	1, 277	0.001	0.04
Sadness	2.09 (1.29)	1.73 (1.34)	4.00	1, 277	0.047	0.01
**CULTURE × REFERENCE EFFECTS**						
Anger	cf. Figure 2	cf. Figure 2	8.02	1, 277	0.005	0.03
Anger at oneself			0.02	1, 277	0.892	0.00
Shame			7.92	1, 277	0.005	0.03
Guilt			8.12	1, 277	0.005	0.03
Sadness			0.00	1, 277	0.996	0.00

*G, Germany; T, Tonga*.

These results support our hypotheses. First, our assumptions on cultural differences were largely corroborated for shame and guilt as well as for anger (but not for sadness; which resonates with findings from Nerb and Spada, 2001). Interestingly, anger at oneself is also reported more often in Germany than in Tonga, even though it shares salient features with shame and guilt, namely that it is directed toward the self (cf. Ellsworth and Tong, 2006). Presumably, it is its aggressive component that renders it less available for Tongan participants. Secondly, and more importantly, the significant interaction effects were in accordance with our expectations of self- and other-serving biases in Germany and Tonga, respectively, in that higher frequency ratings were obtained in the other-referenced version of the questionnaire in Germany, whereas the opposite was true in Tonga.

While the main effects of culture could be due to a range of factors, including memory biases^9^ (Levine et al., 2006), the culture × reference interaction can be interpreted as an effect of culture-specific attribution tendencies on the attribution of emotions.

### Vignettes I: Norm violations

Having established that our samples systematically differ in important aspects of self-concept and emotion assessment, we went on to test whether these differences are reflected in subjects’ appraisal patterns and whether the latter, in turn, lead to different emotional responses, as is predicted by the universal contingency hypothesis (Ellsworth, 1994).

#### Cultural differences in appraisal and emotion

First, we examined whether appraisal and emotion ratings indeed differed between cultures for the chosen scenarios, by subjecting the vignettes data to repeated-measures MANOVAs. While differences between scenarios were expected to occur, these are not interesting *per se*, but rather only in interaction with culture. Scenario main effects will therefore be reported, but not analyzed in detail.

The scenarios of triplet 1 all explicitly described events with negative outcomes caused by another person. We therefore expected a *general* tendency to ascribe more responsibility to others than to self or circumstances in both cultures; accordingly anger should be the prevailing emotional response. However, culture-specific attribution tendencies should also cause differences in the *relative* intensities of responsibility ascription to the three sources (self/other/circumstances) and corresponding differences in additional emotions related to self and circumstances. Aside from the shared core characteristics, the scenarios of triplet 1 systematically varied the cultural salience of the norm violated (GN specific for Germany, TN specific for Tonga, and SN shared by both). We therefore also expected considerable culture × scenario interactions.

Multivariate analysis of the *appraisal data* for this triplet detected significant effects of culture [*F*(4, 217) = 17.62, *p* < 0.001, partial η^2^ = 0.25], scenario [*F*(8, 213) = 14.42, *p* < 0.001, partial η^2^ = 0.35], and culture × scenario [*F*(8, 213) = 11.38, *p* < 0.001, partial η^2^ = 0.30]. Univariate tests demonstrated that overall cultural differences were limited to ratings of other- and self-responsibility (Table 2). As expected, the degree of other-responsibility is rated significantly higher in Germany than in Tonga, whereas Tongans ascribe more responsibility to self than Germans do.

**Table 2:** **Univariate effects of (a) appraisal dimensions and (b) emotional responses for triplet 1 (GN, SN, and TN)**.

	*M* *(SD)_G_*	*M* *(SD) _T_*	*F*	*df*	*p*	*part*. η^2^
**APPRAISAL DIMENSIONS**						
**Culture effects**						
Damage	2.83 (0.76)	2.69 (1.11)	1.42	1, 220	0.235	0.01
Other-responsibility	2.79 (0.74)	1.99 (0.90)	41.01	1, 220	<0.001	0.16
Self-responsibility	0.73 (0.66)	1.53 (1.08)	25.10	1, 220	<0.001	0.10
Responsibility of circumstances	1.47 (0.82)	1.73 (0.96)	0.79	1, 220	0.377	0.00
**Culture × scenario effects**						
Damage	cf. Figure 3A	cf. Figure 3A	16.76	2, 440	<0.001	0.07
Other-responsibility			3.68	2, 440	0.030	0.02
Self-responsibility			9.30	2, 440	<0.001	0.04
Responsibility of circumstances			17.06	2, 440	<0.001	0.07
**EMOTIONAL RESPONSES**						
**Culture effects**						
Anger	2.80 (0.62)	2.67 (0.91)	1.04	1, 269	0.308	0.00
Shame	0.59 (0.72)	1.61 (1.07)	77.47	1, 269	<0.001	0.22
Guilt	0.51 (0.67)	1.92 (1.14)	137.19	1, 269	<0.001	0.34
Sadness	1.78 (0.92)	2.67 (0.94)	46.71	1, 269	<0.001	0.15
**Culture × scenario effects**						
Anger	cf. Figure 3B	cf. Figure 3B	20.52	2, 538	<0.001	0.07
Shame			1.58	2, 538	0.207	0.01
Guilt			0.72	2, 538	0.482	0.00
Sadness			2.92	2, 538	0.056	0.01

**Figure 3:**
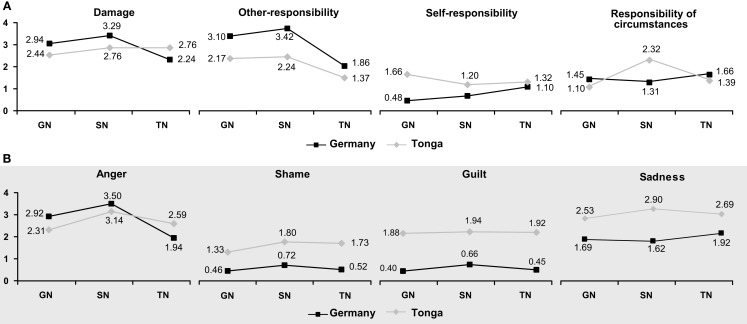
**Intensity ratings of (A) appraisal dimensions and (B) emotional responses for triplet 1 as a function of culture and scenario**.

The culture × scenario interaction, on the other hand, was due to differences in ratings on all four appraisal dimensions (Figure 3A). In particular, the assessment of damage switched between cultures with the cultural salience of the violated norm (higher in Germany for the German norm, higher in Tonga for the Tongan norm). It is also interesting to note that circumstances were most explicitly considered by our Tongan participants in the ballgame scenario (SN), where more than two people are interacting.

Multivariate analysis of the *emotion ratings* for the first triplet of scenarios also showed significant effects of culture [*F*(4, 266) = 43.23, *p* < 0.001, partial η^2^ = 0.39], scenario [*F*(8, 262) = 16.40, *p* < 0.001, partial η^2^ = 0.33], and culture × scenario [*F*(8, 262) = 6.75, *p* < 0.001, partial η^2^ = 0.17]. Subsequent univariate analyses (Table 2) indicated that Tongans and Germans differed in their ratings of shame, guilt, and sadness, with Tongans displaying higher values on all three variables. The culture × scenario interaction was strongly pronounced for anger, indicating that anger intensity was higher when a culturally salient norm had been violated (Figure 3B).

Overall, these results support our hypotheses. Emotional responses to norm violations depend on the salience of the norm (i.e., the perceived degree of damage) and on the sources to which responsibility is ascribed. As Germans predominantly ascribe responsibility to others, they also predominantly respond with anger. Tongans, on the other hand, also consider other sources and give higher ratings for other emotions.

#### Appraisal as mediator of cultural differences

In order to combine the results on appraisal and emotion data reported above within the same model, we conducted additional analyses of moderator and mediator effects, as originally described by Baron and Kenny (1986) and later adapted to within-subjects designs (Judd et al., 2001) and multiple mediator models (Preacher and Hayes, 2008; for a detailed description, see Appendix). This allows for a direct test of our main hypothesis, namely that cultural differences in emotional responses to the different scenarios are mediated by appraisal. In order to facilitate interpretation, we will focus on the emotion of principal interest (i.e., anger) and on the two scenarios that depicted culture-specific norm violations and accordingly yielded clear cultural differences in event appraisal and emotional responses (i.e., scenarios GN and TN).

**Figure 4:**
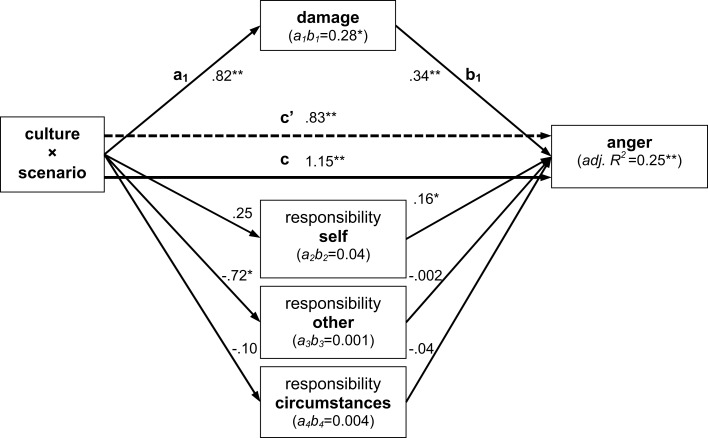
**Results of mediated moderation analyses combining the approaches of Judd et al. (2001) and Preacher and Hayes (2008) for scenarios TN and GN from triplet 1**. The moderating effect of culture was found to be partially mediated by perceived damage, but not via the responsibility dimensions. Direct path coefficients (*a*_*i*_, *b*_*i*_, *c*, *c*′) are noted at their respective arrows; indirect effects (*a*_*i*_*b_i_*) are indicated inside the mediator boxes; proportion of explained variance is indicated for the dependent variable anger. All values reflect normal-theory based testing, but were verified by additional randomization analyses with 10,000 bootstraps. Single asterisks indicate 0.0001 ≤ *p* < 0.05, double asterisks *p* < 0.0001.

Results of the Preacher and Hayes (2008) method applied to appraisal and anger difference scores of scenarios TN and GN demonstrated partial mediation of the moderating effect of culture on anger: that is, while the direct effect (*c*′) remained significant when all potential mediators were simultaneously considered, it was substantially reduced by the significant indirect effect (*a*_*i*_*b_i_*). These results are depicted in Figure 4. They corroborate our interpretation of the results presented in the preceding section: it appears that cultural differences in anger intensity are primarily mediated by differences in the appraisal of damage. However, this effect is not complete as demonstrated by the still significant direct effect of culture × scenario on anger, and this indicates that other appraisal dimensions may be relevant for explaining cultural differences in anger ratings, or that other processes besides the appraisal of the event may affect emotional responses.

### Vignettes IIa: Self vs. other

The scenarios of triplet 2 all described cases in which a material damage occurs that also bears on social relations (gift or party), but they were construed so as to systematically vary causation (by other in OC, by self in SC, and by circumstances in CC). However, whereas the first two scenarios were interpreted in a similar way in the two cultures, this did not hold for the CC scenario. Recall that the CC scenario described a party canceled due to a sudden storm. While weather conditions were regarded as circumstantial in Germany, they were attributed to God – and thus personally (i.e., to other) – by a considerable proportion of our Tongan participants. Both the response patterns and the explanations provided clearly indicated this. The CC scenario was therefore analyzed separately and will be reported in the next section.

#### Cultural differences in appraisal and emotion

With regard to the other- and self-caused events, we expected damage to be assessed similarly across scenarios and cultures (and in fact, the damage occurring is nearly identical in both scenarios). Ascription of responsibility, on the other hand, should depend on the source of causation upon which the respective scenario focuses. Besides this *general* tendency, we further expected Tongans to ascribe responsibility more evenly and, in particular, take circumstances into account more readily in the other-caused scenario than Germans. The variation in causation should also trigger different emotional responses: anger in the other-caused scenario, and shame and guilt in the self-caused scenario. Although anger at oneself is rarely made the subject of discussion in this field (for an exception, see Ellsworth and Tong, 2006), people frequently become angry following negative events caused by themselves. In triplet 2, we therefore included anger at oneself in the list of emotions, and we assumed that results would be largely similar to those for shame and guilt.

**Figure 5:**
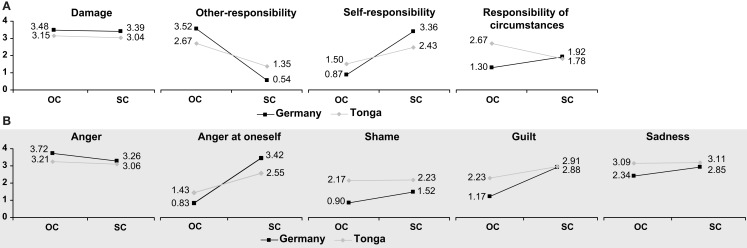
**Intensity ratings of (A) appraisal dimensions and (B) emotional responses for the other-caused (OC) vs. self-caused (SC) scenario as a function of culture and scenario**.

Multivariate analysis of the *appraisal data* showed significant effects of culture [*F*(4, 232) = 4.60, *p* = 0.001, partial η^2^ = 0.07], scenario [*F*(4, 232) = 65.38, *p* < 0.001, partial η^2^ = 0.53], and culture × scenario [*F*(4, 232) = 17.06, *p* < 0.001, partial η^2^ = 0.23]. Subsequent univariate analyses (Table 3) showed intercultural differences pertaining to damage ratings and to ratings of responsibility of circumstances: our German participants rated damage slightly higher than Tongans, who in turn rated the influence of circumstances higher than Germans. The culture × scenario interaction was due to differences in all three dimensions of responsibility ascription (Figure 5A).

**Table 3:** **Univariate effects of (a) appraisal dimensions and (b) emotional responses for the other-caused (OC) vs. self-caused (SC) scenario**.

	*M* *(SD)_G_*	*M* *(SD) _T_*	*F*	*df*	*p*	*part*. η^2^
**APPRAISAL DIMENSIONS**						
**Culture effects**						
Damage	3.42 (0.91)	2.99 (1.18)	4.78	1, 235	0.030	0.02
Other-responsibility	2.01 (0.64)	1.92 (1.05)	0.04	1, 235	0.852	0.00
Self-responsibility	2.13 (0.69)	1.94 (0.76)	1.57	1, 235	0.211	0.01
Responsibility of circumstances	1.58 (0.99)	2.25 (1.30)	11.71	1, 235	0.001	0.05
**Culture × scenario effects**						
Damage	cf. Figure 5A	cf. Figure 5A	0.01	1, 235	0.909	0.00
Other-responsibility			36.85	1, 235	<0.001	0.14
Self-responsibility			31.12	1, 235	<0.001	0.12
Responsibility of circumstances			31.92	1, 235	<0.001	0.12
**EMOTIONAL RESPONSES**						
**Culture effects**						
Anger	3.47 (0.81)	3.05 (1.19)	6.62	1, 252	0.011	0.03
Anger at oneself	2.12 (0.77)	1.97 (1.08)	0.97	1, 252	0.325	0.00
Shame	1.19 (1.05)	2.21 (1.13)	33.33	1, 252	<0.001	0.12
Guilt	2.03 (0.97)	2.58 (1.10)	11.68	1, 252	0.001	0.04
Sadness	2.57 (1.04)	3.05 (0.98)	9.35	1, 252	0.002	0.04
**Culture × scenario effects**						
Anger	cf. Figure 5B	cf. Figure 5B	3.69	1, 252	0.056	0.01
Anger at oneself			34.76	1, 252	<0.001	0.12
Shame			6.10	1, 252	0.014	0.02
Guilt			14.41	1, 252	<0.001	0.05
Sadness			5.10	1, 252	0.025	0.02

As predicted, appraisal patterns followed the variation in causation, and Tongans ascribed responsibility more evenly: in the other-caused scenario they ascribed it less to others but more to self than Germans; in the self-caused scenario, this pattern switches (this finding will be taken up below in the section on interdependent responsibility ascription). Interestingly, Tongans do consider circumstances much more for negative events caused by others than by self, and this is perfectly consistent with the previously observed pattern of a reversed self-serving bias (Bender et al., 2006).

Multivariate analysis of the *emotion data* also revealed significant effects of culture [*F*(5, 248) = 11.03, *p* < 0.001, partial η^2^ = 0.18], scenario [*F*(5, 248) = 46.85, *p* < 0.001, partial η^2^ = 0.49], and culture × scenario [*F*(5, 248) = 8.08, *p* < 0.001, partial η^2^ = 0.14]. Univariate tests of these effects (Table 3) indicated intercultural differences for four of the five emotions: for anger, shame, guilt, and sadness. Corresponding to the results for the first triplet of scenarios – and corroborating our hypothesis – Tongans displayed higher values for shame, guilt, and sadness, while anger ratings were higher in Germany. Finally, differences in emotional responses across scenarios were less pronounced in Tonga than in Germany (as were differences in appraisal). Culture × scenario interaction effects were significant for anger at oneself, shame, guilt, and sadness, but only marginally significant for anger (Figure 5B). The findings indicate that Tongans respond more strongly to the outcome of the event itself (i.e., the damage caused) than to its causes, whereas emotional responses among the German sample differentiated much more with regard to responsibility.

It is also worth noting that in both cultures, the switch in causation, and accordingly in the ascription of responsibility, across scenarios is reflected most precisely by anger at oneself – in fact, more precisely than by shame or guilt, and particularly more than by anger.

#### Mediated moderation analyses

As for the first vignettes triplet, we went on to test whether appraisal patterns could conceivably mediate the moderating effect of culture on participants’ emotional response patterns to the two scenarios. As for scenarios TN and GN, we used difference scores for emotional responses and appraisal dimensions to take into account our within-subjects design, and conducted multiple mediator analyses as proposed by Preacher and Hayes (2008) on these scores. Separate analyses were conducted for anger and anger at oneself as dependent variables.

**Figure 6:**
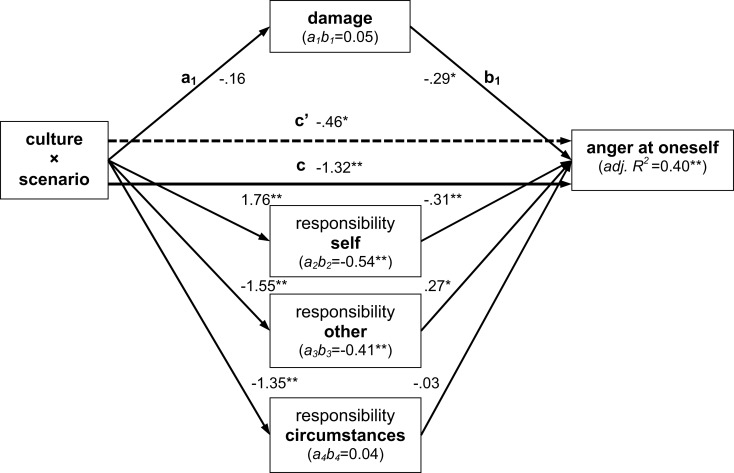
**Results of mediated moderation analyses for anger at oneself across scenarios SC and OC from triplet 2**. Results indicate partial mediation of the moderating effect of culture by perceived self- and other-responsibility; all conventions as in Figure 4.

In accordance with the results presented in the preceding section, analyses of *anger* scores indicated an only marginally significant moderating effect of culture (*c* path, *p* = 0.071). In turn, none of the indirect effects via the appraisal dimensions were significant either (all *p* ≥ 0.19). Results for *anger at oneself* as the dependent variable, on the other hand, indicated significant indirect effects via self- and other-responsibility, both if tested based on normal theory and following 10,000 bootstraps (detailed results in Figure 6). Again, mediation of the moderating effect of culture was not complete, as indicated by the still significant *c*′ path. One reason why indications of self-anger appear to be more sensitive to experimental variation than other-anger could be that the scenario itself involves the self as cause in both the OC and SC version – after all, it is Tom (the person himself) who lets the gift fall down.

These findings indicate that cultural differences in the expression of anger at oneself, an emotion rarely studied in the context of appraisal theory, are partly mediated by differences in the attribution of responsibility for negative events to oneself and others. It remains to be tested whether the lack of moderation and mediation effects for other-anger observed across the present scenarios generalizes to other cases where responsibility of oneself or another person appears as the dominant factor of a negative event.

It is interesting to note that for both anger at oneself (cf. Figure 6) and anger, culture moderated the effect of scenarios SC and OC on ratings of responsibility of circumstances, but that this appraisal dimension in turn did not have any effect on either of the two dependent variables. This suggests that external circumstances for a negative event are not translated into any form of anger, in accordance with the idea that negative events caused by circumstances beyond one’s control should elicit sadness rather than anger.

### Vignettes IIb: Acts of god vs. circumstances

As mentioned above, interpretations of scenario CC (in which the party had to be canceled because of a sudden storm) diverged across cultures. While the storm was regarded as circumstantial by our German participants, the «person» responsible for the storm triggered ascription of responsibility to other among many Tongan participants. Treating the CC scenario thus separately obviously precludes the sort of moderation and mediation analyses employed above to test the hypothesis of culture-specific emotional responses as being mediated via appraisal patterns. Analyses of cultural differences in appraisal and emotions are nevertheless reported for completeness.

Multivariate analysis of the *appraisal data* for this scenario showed a significant effect of culture [*F*(4, 241) = 12.27, *p* < 0.001, partial η^2^ = 0.17] which was due to differences in ratings of all four appraisal dimensions (Table 4). While Germans rated damage and responsibility of circumstances higher, Tongans did so for other- and self-responsibility.

**Table 4:** **Univariate effects of (a) appraisal dimensions and (b) emotional responses for the circumstances-caused scenario (CC)**.

	*M* *(SD)_G_*	*M* *(SD) _T_*	*F*	*df*	*p*	*part*. η^2^
**APPRAISAL DIMENSIONS**						
Damage	3.46 (0.90)	2.77 (1.50)	17.53	1, 244	<0.001	0.07
Other-responsibility	0.71 (1.26)	1.60 (1.49)	19.02	1, 244	<0.001	0.07
Self-responsibility	1.04 (1.23)	1.58 (1.50)	7.07	1, 244	0.008	0.03
Responsibility of circumstances	3.09 (1.28)	2.02 (1.64)	25.55	1, 244	<0.001	0.10
**EMOTIONAL RESPONSES**						
Anger	3.38 (1.07)	2.62 (1.57)	16.28	1, 259	<0.001	0.06
Anger at oneself	1.28 (1.37)	1.49 (1.56)	0.89	1, 259	0.346	0.00
Shame	1.49 (1.27)	2.66 (1.37)	31.97	1, 259	<0.001	0.11
Guilt	1.26 (1.35)	2.47 (1.53)	29.48	1, 259	<0.001	0.10
Sadness	2.93 (1.17)	2.79 (1.44)	0.56	1, 259	0.454	0.00

Multivariate analysis of the *emotion data* again revealed a significant effect of culture [*F*(5, 255) = 13.72, *p* < 0.001, partial η^2^ = 0.21], which was due to differences in ratings of anger, shame, and guilt (Table 4). Germans displayed higher ratings of anger, while Tongans gave higher ratings for shame and guilt. Ascribing responsibility to others as much as to self, Tongans also responded with anger to a degree similar to shame and guilt. Interestingly, the angry responses of our German participants do not consistently follow from their appraisal pattern: although their ascription of responsibility to other is far outweighed by ascription to circumstances, they still indicate anger as their most intensive emotion, apparently triggered by their assessment of damage.

## Discussion

Overall, we found corresponding cultural differences in the three types of variables under scrutiny: in the prevailing self-concept, in the relevant cognitive determinants of emotions, and in the emotional responses themselves. Germans appear to have a less interdependent self-concept than Tongans. In general, they also tend to appraise more damage, ascribe more responsibility to others, and accordingly respond with anger more often or more intensely than Tongans. Tongans, in turn, ascribe more responsibility to self and circumstances, which results in higher ratings for shame and guilt as well as sadness, respectively. This is largely confirmed by an analysis of how moderating effects of culture on anger are mediated by the appraisal of damage and responsibility.

While the above summary adequately describes the overall tendencies, three exceptions need to be emphasized. First, assessing damage and responding angrily depends on the cultural salience of the eliciting event. The violation of norms, for instance, gives rise to anger in both cultures, but only if the respective norm is generally agreed upon. When switching between the Tongan-specific norm violation and the German-specific norm violation, the appraisal patterns switch accordingly between cultures. In this case, the cultural impact on anger is mediated by the appraisal of damage (cf. Figure 4).

Second, while Tongans – at least in general – tend to ascribe more responsibility to self and circumstances and less to others than Germans, this pattern switches in cases in which Germans ascribe responsibility to these sources to a large extent (e.g., in the self-caused and circumstances-caused scenario, respectively). In other words: Tongans ascribe responsibility more moderately and more evenly to the three potential sources of causation than Germans (who tend to focus on one single source for each scenario). These tendencies are reflected in emotional responses, which are generally more evenly spread in Tonga and more focused in Germany.

This pattern could be a side effect of an interdependent self-concept, according to which people are not individually responsible, but rather collectively responsible. Concurring evidence is reported by Menon et al. (1999) and Morris et al. (2001), who found that members of collectivistic cultures tend to ascribe responsibility to a different entity, namely to groups instead of individuals (and see Mesquita and Markus, 2004). It is also interesting to note that Tongans consider circumstances less readily when the damage is caused by self than by others, while the opposite holds for Germans. Consistent with previous findings (Bender et al., 2006), this pattern corresponds to a self-serving bias in Germany and a reversed self-serving bias in Tonga.

Third, the general ascription pattern is also reversed in the circumstances-caused scenario. Apparently, the storm responsible for the canceled party was interpreted by German participants as caused by circumstances, while this was only partly the case in Tonga, where often a personal agent was also held responsible. Yet, in this case, the reason for the divergence is more interesting than the divergence itself, as it indicates that cultural differences occur even with regard to how causes are categorized. While respective effects of culture on causal categorization have been explored in various domains (e.g., Malinowski, 1948; Bender, 2001; O’Barr, 2001; Bang et al., 2007; Froerer, 2007; Astuti and Harris, 2008; Nerb et al., 2008; Beller et al., 2009b; Bender and Beller, 2011a), its impact on the cognitive determinants for emotion elicitation clearly deserves more attention in future research (cf. Ellsworth, 1994).

Taken together, these experimental data and the data collected using the emotion frequency questionnaire suggest that Tongans emphasize and experience self-related negative emotions more strongly than Germans. Socially disruptive emotions, on the other hand, and particularly anger, appear less frequently in Tonga than in Germany. As we have detailed above, this difference in occurrence is linked to differences in the cultural evaluation of anger. Do we therefore have to assume that our data are determined by social desirability? We do not believe this to be the case, at least not extensively, for three reasons: first, although cultural norms in Tonga restrict the portrayal of socially disruptive emotions in public, they do not prohibit talking about such emotions or admitting to them in an abstract way. Second, those of our Tongan participants who had to rate the frequency of their own emotions actually indicated *more* anger than those who rated others’ emotions. And third, the response patterns in our experimental setting changed consistently with the eliciting determinants, as predicted. In other words, Tongans *had to* indicate less anger simply because they rated damage less strongly and ascribed less responsibility to others. Nevertheless, the results of the frequency questionnaire do indicate that estimations about emotions are biased – otherwise, they should not differ with regard to self- or other-referencing. However, these differences are in accordance with more general attribution tendencies in each culture and thus more likely reflect these tendencies than socially motivated response tendencies.

On the other hand, we can (and need) not rule out that the cultural evaluation of specific emotions may affect the appraisal tendencies to a similar extent as the cognitive appraisal affects emotion elicitation. Such an assumption is supported by coherence models of cognition and emotion (e.g., Thagard, 2000; Nerb and Spada, 2001; Thagard and Nerb, 2002) as well as by Scherer and Brosch’s (2009) proposition that cultural values may encourage certain types of appraisal tendencies (see also Mesquita and Walker, 2003). A similar argument was put forward in Bender et al. (2007a): they observed that, in cultural contexts with a strong disapproval of anger, factors that would give rise to anger tend to be discounted.

Clearly, the methods we adopted restrict the range for generalization. Our participants were recruited from only one high school in each country, and the vignettes were constructed from situations familiar to this part of the population. However, the prime goal of our study was not to collect a representative sample of typical emotions and their frequency of occurrence in the two cultures, but to scrutinize the cognitive determinants and processes that elicit specific emotional responses and to identify the factors that may affect those processes. Given this goal, restrictions in sample and vignettes appear not to be too severe. More crucial are questions of validity connected to the use of vignettes. The advantage of this kind of studies is their *internal* validity: they allow us to interpret – with considerable confidence – differences in emotional responses as effects of the experimental manipulation in appraisal dimensions. With regard to *external* validity, however, vignette studies are disadvantaged, if one is interested in “real” emotions. Do vignette studies really assess emotional responses and their cognitive determinants, or do they assess folk-psychological theories about how emotions are elicited? Importantly, we *are* interested in folk-psychological theories about how emotions are elicited. In fact, our main concern was to collect data that speak to questions of how members of different cultures process information that is related to emotions.

In addition, however, we believe that our data *also* speak to emotion elicitation itself. While we cannot entirely compensate for the disadvantage mentioned above, convergent findings from two different sources encourage us to believe that we did get as close to the “real” emotions as is possible in this kind of research. First, the findings presented here replicate findings from previous research in Tonga on related topics (Bender, 2002; Beller and Bender, 2004; Bender et al., 2006, 2007b; Beller et al., 2009a). And second, our inferences on the cultural framing of emotions in Tonga are further backed up by anthropological research on emotional responses in daily life and on the social structure and cultural values pertaining to emotions (e.g., Morton, 1996; Bender et al., 2007a). Furthermore, the converging evidence gathered by appraisal theoretic research is increasingly regarded as supporting the assumption that folk-psychological ideas are in fact a good indication of how emotions are elicited (Scherer et al., 2001).

More generally, our results on self-concept and its impact on cognitive determinants of emotions are consistent with findings from research on attribution styles (e.g., Morris et al., 1995, 2001; Choi and Nisbett, 1998). In particular, the patterns for ascribing responsibility follow the cultural differences in self-concept in the predicted way. For instance, one of our pervasive findings is that Tongans emphasize and experience self-related negative emotions such as shame and guilt more strongly, but socially disruptive emotions such as anger less strongly than Germans. An analogous variation was reported by Kitayama et al. (2006) for Japan and the US, where, again, prevalence of socially engaging vs. disengaging emotions depended on which type of self-concept was favored in the respective culture^10^.

Yet, our study does not simply attest such cultural variation, but attempts to provide an explanation for it on a more fine-grained level by integrating the observed cultural differences in attribution tendencies with a cognitive, appraisal-theoretical account. This allows us to account for cultural differences in emotional responses not only as a result of culture-specific concepts and values, but as mediated by differences in appraisal patterns.

In doing this, our study also contributes to appraisal theories, as it identifies how culture-specific appraisal patterns are affected by differences in attribution styles. In general, the observed correspondence between cognitive determinants and emotional responses reflects appraisal-theoretical findings (e.g., Ellsworth and Smith, 1988; Smith et al., 1993; Roseman et al., 1995, 1996; Scherer, 1997a). Despite the label as the “Friendly Islands,” people in Tonga seem to experience anger in ways similar to Bender et al. (2007a) and nearly as often as people in Germany. Even the cognitive determinants eliciting anger are largely the same: anger is elicited by an appraisal of damage and other-responsibility, whereas self-responsibility co-varies with shame and guilt. For most cases, the effect of scenarios on appraisal and emotional responses was stronger than the effect of culture.

But beyond these general similarities, remarkable differences can also be discerned, at least in terms of detail: although the same set of cognitive determinants is accountable for the elicitation of anger, these do not appear to be equally important across cultures. While in both cultures the assessment of damage contributes more strongly to angry responses than does ascribing responsibility to others, this tendency is even stronger in Tonga. Here, the outcome of an event seems to determine the resulting emotion more than the assessment of who is responsible (this is particularly apparent in the second triplet, where causation was systematically varied). This interpretation is consistent with the more balanced patterns of both responsibility ascription and emotional responses in Tonga. Across all scenarios (except the ambivalent CC scenario), sadness is significantly stronger in Tonga than in Germany and, in four out of six scenarios, it is even stronger than anger. These findings concur with previous work by Kuppens et al. (2003), who found empirical evidence for a contingent association between cognitive determinants and the emotional response, rather than a strong link between the two. Applied to our findings, this would entail that responsibility is less strongly focused upon and subsequently less accessible in the Tongan culture (for additional support see Bender et al., 2007a); consequently, it offers less predictive validity for certain emotions as compared to Western cultures.

We also found evidence for a necessary differentiation with regard to anger itself. Most appraisal theories predict anger as a response to negative events caused by other people, and shame and guilt as responses to negative events caused by oneself. Our data from both the frequency and the vignettes questionnaires show that anger can occur in response to self-caused events – not as often in Tonga as in Germany, but nevertheless quite frequently in both cultures, thus replicating the findings of Ellsworth and Tong (2006) in a non-Western culture. In fact, anger at oneself even reflects changes in causation and ascription of responsibility much more precisely than the self-related emotions shame and guilt. Conversely, and again similar to Ellsworth and Tong’s (2006) findings, we did not find considerable differences in the relationship of guilt and shame across cultures (cf. Fontaine et al., 2006). In our scenarios, guilt is always nearly as high as or even higher than shame in both cultures, with nearly parallel patterns. In addition, both guilt and shame are consistently higher in Tonga than in Germany.

To conclude, our findings supplement previous evidence that cultural concepts and preferences have an impact on attribution tendencies. In Germany and Tonga, this impact could be demonstrated for attributing emotions to others, for the relative focus on specific appraisal dimensions, and for patterns of ascribing responsibility. Even more importantly, these same cultural concepts and preferences also affect subsequent emotional responses, in that they emphasize personal responsibility differently. Our experimental evidence for such an impact therefore supports a cognitive explanation for cultural differences in emotions that is more differentiated than previous accounts. In addition, though, our findings also indicate that other culture-specific factors besides self-concept are at work in shaping cognitive appraisals of causation and responsibility (cf. Beller et al., 2009a,b). Identifying and assessing these additional factors requires – and deserves – more research in the future.

## Conflict of Interest Statement

The authors declare that the research was conducted in the absence of any commercial or financial relationships that could be construed as a potential conflict of interest.

## Figures and Tables

**Figure A1:**
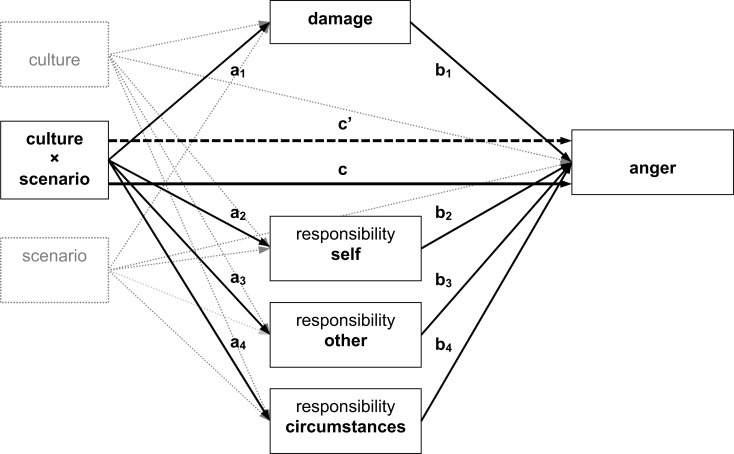
**Basic model of direct and indirect effects on anger**. The model assumes that the moderating effect of culture (i.e., the culture × scenario interaction) on anger is mediated via the appraisal dimensions of damage and responsibility (of self, other, and circumstances). Using difference scores in order to take into account our within-subjects design, we effectively tested the simplified model printed in black. Paths *a*_*i*_ designate the direct effects of the culture × scenario interaction; paths *b*_*i*_ are the direct effects of the mediators on the dependent variable anger. Path *c* is the total effect of the culture × scenario interaction on anger; path *c*′ is the direct effect remaining once the indirect effects via the potential mediator variables are taken into account. A significant *c* path in conjunction with a non-significant *c*′ path indicates complete mediation, but the analyses we employed also permit direct significance testing of the *a*_*i*_*b_i_* paths.

## Footnotes

^1^The definition of *shame/guilt* as largely self-caused emotion is prototypical, but not exhaustive, and may itself depend on cultural context. Research on Chinese, for instance, indicates that a general category of shame may subsume both self-shame and other-shame. Other-shame describes the types of shame-like feelings one may feel in response to another person’s actions (Li et al., 2004). For pointing us to this reference, we thank one of our anonymous reviewers.

^2^Of course, like most Pacific island countries, Tonga was subject to European colonial endeavors in the nineteenth and twentieth centuries, and is nowadays integrated into globalized political and economic networks, predominantly with Western countries, but also with China and other East Asian countries. Nevertheless, the fundamental cultural values and social structure have only partly been affected by these cultural influences (e.g., van der Grijp, 1993; Morton, 1996; Evans, 2001; Lee, 2003).

^3^This lack of difference on the independence subscale is in line with the notion that the two dimensions *independence* and *interdependence* are not mutually exclusive, and thus need not be expressed antithetically across cultures (for respective evidence, see Oyserman et al., 2002). In fact, a strong (cultural) focus on interdependence is not *per se* incompatible with the (individual) desire to also keep one’s independence whenever possible. This is reflected in the sketch of cultural values, which express dislike both for disrespectful behavior and for events that curb one’s personal freedom.

^4^In theory, we had a choice between high school students, university students, and older people. Older people are not familiar with questionnaires and rating scales, and the majority of Tongan university students are enrolled overseas. High school students are more familiar with questionnaires than older people and, in addition, are more representative of the whole population than university students – with the exception of their age. However, competence related to a particular age is not required for experiencing emotions, but only for the restriction of emotion. Juvenile participants, one may assume, could be more willing to admit to emotions to which adults might not admit. If we still find cultural differences between the two groups, they should accordingly have even more weight.

^5^The following items were excluded: for interdependence “I should take into consideration my parents’ advice when making education/career plans” and “Even when I strongly disagree with group members, I avoid an argument,” and for independence “I’d rather say ‘No’ directly, than risk being misunderstood” and “I feel comfortable using someone’s first name soon after I meet them, even when they are much older than I am.” Apart from being inconsistent with the rest of the subscale, two of them (the first and the last) didn’t make sense in the Tongan context and/or to the pupil sample with whom we worked.

^6^As we are particularly interested in correlations of ascriptions to self and other, we used the same content for these two scenarios, varying the source of causation only, despite the risk of carry-over effects. The third scenario differed in content, which might render comparability difficult, but – as we will see below – it had to be analyzed separately in any case.

^7^The remainder consisted of indignation, helplessness, dislike, pity, regret, disappointment, and contempt, and was supplemented by an open question. Please note that, although indignation would have been particularly interesting – as it constitutes a sub-case of anger related to norm violations – this is the case in German only; in Tongan, we didn’t find a term close enough in meaning to warrant its inclusion in the subsequent analyses (this lack of lexical correspondence does not imply, of course, that the feeling itself could not be recognized; cf. Sauter et al., 2011).

^8^Of the emotion terms under investigation, only “shame” was not entirely similar in the two languages. More strongly than the German term *Scham*, which is sufficiently close to the English translation, the Tongan word $m\bar{a}$ also includes notions of shyness and embarrassment. However, as “shame” is the prototypical emotional state referred to with this term, and $m\bar{a}$ its only gloss, we decided to use it nonetheless, thus hazarding the possible consequence that lower frequencies of reporting *Scham* in Germany could be simply due to the more restricted meaning of this term, as compared to $m\bar{a}$ in Tonga. The inclusion of guilt in the analyses partly serves to compensate for this drawback.

^9^In principle, these main effects could also be due to culture-specific patterns in responding to rating scales. To control for such tendencies (as advised by one of the anonymous reviewers), we conducted a second MANOVA, based on centered means that were calculated by subtracting the overall mean across all emotions of each cultural group (*M*_*G*_ = 1.89, *SD*_*G*_ = 0.73; *M*_*T*_ = 1.69, *SD*_*T*_ = 0.71) from the emotion-specific individual responses. This analysis produced almost identical results and still obtained an overall main effect of culture [*F*(5, 273) = 16.44, *p* < 0.001, partial η^2^ = 0.23], thus speaking against response tendencies as the only relevant factor.

^10^In a similar vein, the Tongan pattern of emphasizing negativity of the self in relation to others, and the reversed pattern among our German participants resonate with findings on the cross-cultural variation in self-serving bias or self-enhancement vs. criticism (e.g., Kitayama et al., 1997; Heine et al., 1999).

## Appendix

### Appraisal as mediator of cultural differences

Our analyses of moderation and mediation effects is based on procedures originally described by Baron and Kenny (1986) and later adapted to within-subjects designs (Judd et al., 2001) and multiple mediator models (Preacher and Hayes, 2008).

Previously published methodological work has examined the analysis of moderator and mediator effects in within-subject designs (Judd et al., 2001) and the combined analysis of multiple mediators (Preacher and Hayes, 2008). However, to the best of our knowledge the simultaneous consideration of these two problems has not been investigated in detail. We therefore combined the two published approaches by first calculating difference scores for the dependent variable anger, as well as sum and difference scores for all mediator variables (damage, responsibility of self/other/circumstances), as suggested by Judd et al. (2001). This approach incorporates the main effect of the different scenarios on anger ratings in the difference scores which function as the dependent variable. Testing for effects of culture on these difference scores thus amounts to testing the interaction of culture and scenario on anger ratings.

Figure A1 shows the complete model of direct and indirect effects (Preacher and Hayes, 2004) to be tested. This model combines (1) a moderation model testing whether the interaction of culture and scenario has a significant effect on anger ratings, and (2) a mediation model testing whether effects of scenario, culture, or their interaction on anger ratings are mediated via the four appraisal dimensions of interest. The simplified model resulting from the use of difference scores is highlighted in black.

The Judd et al. (2001) approach to within-subject analysis of moderator and mediator effects does not in itself provide for testing combinations of these two processes: mediated moderation and moderated mediation (Baron and Kenny, 1986; Muller et al., 2005). However, as it simplifies the interaction between the independent variable and the potential moderator to a simple effect of the moderator on difference scores, standard mediator analyses can be used on this “camouflaged” moderator effect to test our principal hypothesis, namely whether cultural differences in anger ratings are mediated by differences in event appraisal. We employed the procedure proposed by Preacher and Hayes (2008), which allows for the simultaneous testing of several potential mediators, both based on normal distribution theory and using re-sampling techniques. Whereas the traditional Baron and Kenny (1986) approach rests on a descriptive comparison of the direct paths linking the predictor to the dependent variable (i.e., *c* and *c*′) in the presence or absence of potential mediators, the solution proposed by Preacher and Hayes (2008) allows for a direct significance test of the indirect paths (a_i_b_i_) and thus a more stringent test of the model.

One drawback of these methods is that the combined analysis of appraisal and emotion data across two scenarios exacerbates the problem of missing data: while cases with missing data only rarely exceeded 10% of responses per variable for the analyses reported in the preceding section, combined analysis of all variables would have led to sample attrition of up to 40% in both cultures. We therefore employed multiple imputation as implemented in SPSS/PASW 17 to complete our dataset for the four emotions and the four appraisal dimensions of interest. Standard settings were used and results of the fifth iteration were employed for all subsequent analyses (Tabachnick and Fidell, 2007). In order to assess potential effects of data imputation, we repeated the analyses reported above using the completed dataset. The only difference was that main effects of culture on responsibility of circumstances and on anger are now significant. This is probably due to the increased power with higher *N*. Importantly, it has no negative consequence for the interpretation of our results. For the scenarios of triplet 2, no changes in significance levels were observed when imputed data were used.
